# World Hepatitis Day — July 28, 2019

**DOI:** 10.15585/mmwr.mm6829a1

**Published:** 2019-07-26

World Hepatitis Day, observed each year on July 28, was established to raise awareness and promote understanding of viral hepatitis around the world. The theme of this year’s World Hepatitis Day is “Invest in Eliminating Hepatitis,” underscoring the need to increase commitment for hepatitis response. In 2015, an estimated 257 million persons were living with hepatitis B and 71 million with hepatitis C worldwide (*1*).

Persons who inject drugs are at highest risk for hepatitis C virus (HCV) infection. Globally, an estimated 15.6 million persons aged 15–64 years inject drugs, 52% of whom are HCV-antibody positive (*2*). This issue of *MMWR* features a report on the progress in the country of Georgia toward prevention and detection of HCV infection, and linkage to treatment, of persons with HCV infection who inject drugs (*3*). Georgia’s hepatitis C elimination program, launched in 2015, was recently named the world’s first Centre of Excellence in Viral Hepatitis Elimination by the European Association for the Study of the Liver International Liver Foundation. Access to hepatitis C testing and treatment for persons who inject drugs is critical to achieving elimination in countries where persons who inject drugs account for a significant proportion of HCV infection. Additional information and resources about viral hepatitis are available at https://www.cdc.gov/hepatitis.
